# Thermostability-based binding assays reveal complex interplay of cation, substrate and lipid binding in the bacterial DASS transporter, VcINDY

**DOI:** 10.1042/BCJ20210061

**Published:** 2021-11-09

**Authors:** Connor D. D. Sampson, Cristina Fàbregas Bellavista, Matthew J. Stewart, Christopher Mulligan

**Affiliations:** School of Biosciences, University of Kent, Canterbury, Kent CT2 7NH, U.K.

**Keywords:** lipids, membrane proteins, membranes, molecular interactions, transport

## Abstract

The divalent anion sodium symporter (DASS) family of transporters (SLC13 family in humans) are key regulators of metabolic homeostasis, disruption of which results in protection from diabetes and obesity, and inhibition of liver cancer cell proliferation. Thus, DASS transporter inhibitors are attractive targets in the treatment of chronic, age-related metabolic diseases. The characterisation of several DASS transporters has revealed variation in the substrate selectivity and flexibility in the coupling ion used to power transport. Here, using the model DASS co-transporter, VcINDY from *Vibrio cholerae*, we have examined the interplay of the three major interactions that occur during transport: the coupling ion, the substrate, and the lipid environment. Using a series of high-throughput thermostability-based interaction assays, we have shown that substrate binding is Na^+^-dependent; a requirement that is orchestrated through a combination of electrostatic attraction and Na^+^-induced priming of the binding site architecture. We have identified novel DASS ligands and revealed that ligand binding is dominated by the requirement of two carboxylate groups in the ligand that are precisely distanced to satisfy carboxylate interaction regions of the substrate-binding site. We have also identified a complex relationship between substrate and lipid interactions, which suggests a dynamic, regulatory role for lipids in VcINDY's transport cycle.

## Introduction

The SLC13 transporter family catalyses the transport of dicarboxylates, such as succinate, the tricarboxylate citrate, and sulfate across the plasma membrane of human cells. The five members of the SLC13 family, NaDC1, NaDC3, NaCT, NaS1 and NaS2, share ∼50% identity with each other, and have distinct functional properties in an array of tissues in the human body including the liver, kidney, brain, and placenta [1]. Uptake of SLC13 substrates affects several physiological and pathophysiological processes in humans, ranging from the regulation of oxidative metabolism in hepatocytes and neurons to the secretion of citrate into the urine to prevent kidney stones [1]. In addition, disruption of NaCT activity in liver cancer cells halts their proliferation [2].

The SLC13 transporters are members of the large divalent anion:Na^+^ symporter (DASS) family (TDCB family 2.A.47 [3]), which includes members from all domains of life. The DASS family can be split broadly into two different types; co-transporters that couple substrate transport to a Na^+^ gradient (DASS-C), and exchangers that exchange dicarboxylates across the membrane (DASS-E) [4]. However, DASS-C transporters have been the primary focus of research due to their pharmacological relevance. Functional disruption of DASS transporters in several model organisms has revealed their activity profoundly influences metabolism. Knockdown of the gene encoding the DASS family member, *I'm not dead yet* (INDY), in *Drosophila melanogaster* led to a condition resembling caloric restriction and doubled the lifespan of the fly; a result replicated in the nemotode, *Caenorhabditis elegans* [5, 6]. In addition, disrupting a DASS transporter (NaCT) in a mouse model resulted in resistance to adiposity and insulin insensitivity when fed a high fat diet [7], reinforcing the link to metabolism and unveiling DASS family members as extremely attractive therapeutic targets in the treatment of age-related metabolic diseases.

To develop therapeutics targeting these transporters, a structural understanding of substrate and inhibitor binding is essential. The most comprehensively characterised DASS transporter to date is VcINDY, which shares functional and structural characteristics with the human DASS family members [8–11], making it an excellent structural model. Structural characterisation of VcINDY reveals it to be homodimeric, with each protomer containing two distinct domains; a scaffold domain that contributes all of the dimerisation contacts, and the transport domain that houses the majority of the substrate binding residues, including the signature ‘SNT’ motifs [12, 13]. The SNT motifs are located at the tips of two re-entrant hairpin loops, and form many crucial contacts with the substrate [12, 13]. These re-entrant hairpin loops found in VcINDY are a feature shared by other Na^+^-driven transporters, including; members of the dicarboxylate/amino acid: cation symporter (DAACS) family, for example the SLC1 family and the very well characterised archaeal protein Glt_Ph_ [14, 15]; concentrative nucleoside transporters, for example, the SLC28 family [16, 17]; and the 2-HCT family citrate transporter [18, 19]. Each of these transporters facilitate transport of their cargo across the membrane via an elevator-like mechanism in which the substrate-loaded transport domain translocates vertically through the bilayer, alternately exposing the substrate-binding site from one side to the other [4, 15, 17, 20–22].

DASS transporters from both eukaryotic and prokaryotic sources have now been characterised, revealing variation in the substrate specificity, affinity and transport energetics. Of the human transporters, NaDC1 and NaDC3 are primarily C_4_-dicarboxylate transporters (but have the ability to transport citrate) [23–27], NaCT is primarily a citrate transporter (but retains the ability to transport C_4_-dicarboxylates) [28], and NaS1 and NaS2 are sulfate and selenate transporters [29–31]. Analysis of the substrate range of characterised non-human DASS transporters reveal overall similar substrate specificity with C_4_-dicarboxylates, succinate, fumarate malate being common ligands and/or substrates [8, 32–36]. However, some variation is observed. For example, only some have been shown to interact with the amino acids, such as aspartate and glutamate [24, 27, 33, 34, 37, 38]. Due to the lack of structural insight into ligand binding for this protein family, it is hard to characterise the structural basis for these selectivity variations. Previous characterisation with a limited set of compounds revealed that VcINDY preferentially interacts with, and transports, the C_4_-dicarboxylates, succinate, malate, fumarate and oxaloacetate [8]. However, a thorough systematic analysis of the requirements of a DASS transporter ligand is still required.

Here, using the model DASS co-transporter VcINDY, we have sought to extend the range of compounds that are known to interact with DASS transporters. Using a high-throughput thermostability-based approach, we have probed 71 different compounds and identified 18 compounds that interact with VcINDY, several of which are novel substrates/inhibitors of DASS transporters. Our findings have allowed us to generate a list of rules that define what is required of a small molecule to allow it to bind to VcINDY. In addition, we have, for the first time, examined cation and lipid binding to a DASS transporter and probed how these interactions influence substrate interactions. Our data suggest that VcINDY is able to bind Na^+^ ions in the absence of substrate and this is an *essential* prerequisite for substrate interactions. Furthermore, our data reveal a complex relationship between lipid and substrate interactions in which interactions with specific lipids appear to be modulated by the substrate bound state of the protein. These data suggest a dynamic role for lipid binding in the transport cycle of DASS transporters.

## Materials and methods

### Protein expression and purification

VcINDY was expressed using the MemStar system [39]. Briefly, Lemo21 (DE3) (New England Biolabs) harbouring the expression vector pEThisINDYwt, a modified pET vector with the gene encoding VcINDY expressed in-frame with an N-terminal deca-histidine tag [40], was grown in PASM-5052 media [41] with MemStar modifications in the presence of 100 µg/ml kanamycin, 25 µg/ml chloramphenicol, and 0.25 mM L-rhamnose [39]. Protein expression was induced at a culture OD_600_ of 0.5 by the addition of 0.4 mM isopropyl β-d-1-thiogalactopyranoside (IPTG). Cultures were incubated overnight at 25°C before being harvested and lysed via sonication. Protein was purified as done previously [8]. The insoluble membrane fraction was collected by ultracentrifugation and resuspended in Purification Buffer (PB, 50 mM Tris pH 8, 100 mM NaCl, and 5% glycerol). VcINDY was extracted from the membrane by the addition of 20 mM n-dodecyl-β-d-maltoside (DDM) and insoluble material was removed by ultracentrifugation. DDM-solubilised protein was incubated overnight at 4°C with Talon metal affinity resin (Takara Bio Inc.). The protein-bound resin was washed with 20 column volumes (CV) of PB supplemented with 2 mM DDM and 10 mM imidazole, followed by 20 CV PB supplemented with 2 mM DDM and 20 mM imidazole. Bound protein was eluted through the addition of 1.5 CV buffer containing 2 mM DDM and 10 µg/ml trypsin with a one hour incubation at 4°C, which also resulted in cleavage of the deca-histidine tag. For analysis by size exclusion chromatography (SEC), Talon-purified protein was applied to a Superdex 200 column (GE Healthcare) equilibrated with Purification Buffer supplemented with 2 mM DDM.

### Protein reconstitution and *in vitro* transport assays

The functional reconstitution of VcINDY variants was performed as previously described with some minor modifications [20, 42]. An amount of 25–100 µg of purified protein was diluted into 2 ml Reconstitution Buffer (25 mM Tris, 100 mM NaCl, 5% glycerol, and 3% n-decyl-β-d-maltoside (DM)) and mixed with 8 mg of *E. coli* polar lipid extract (Avanti Polar Lipids). The protein/lipid mixture was incubated for 10 min on ice and rapidly diluted by addition to 65 ml of Inside Buffer (20 mM Tris, pH 7.5, 1 mM NaCl, 199 mM KCl) or detergent was removed by multiple additions of Biobeads (Bio-Rad). Proteoliposomes were collected by ultracentrifugation, resuspended to 8 mg/ml lipid in Inside Buffer, freeze-thawed three times and stored at −80°C.

For *in vitro* transport assays, proteoliposomes were extruded 11 times through a 400 nm filter, collected by ultracentrifugation, and resuspended in Inside Buffer to a final concentration of 80 mg/ml lipid. Transport assays were initiated by addition of proteoliposomes to Reaction Buffer (20 mM Tris, pH 7.5, 100 mM NaCl, 100 mM KCl, 1 µM valinomycin, and 1 µM [^3^H]-succinate (American Radiolabelled Chemicals). Samples collected were quenched by addition of Quench Buffer (20 mM Tris, pH 7.5, 200 mM choline) and proteoliposomes were immobilised on 200 nm nitrocellulose filters (Millipore) via rapid filtration. Filters were washed with Quench buffer and accumulated [^3^H]-succinate was measured by combination of FilterCount Liquid Scintillation cocktail (PerkinElmer) and a Hidex 300SL Liquid Scintillation Counter.

### CPM-based thermostability assay

VcINDY was buffer exchanged into N-[4-(7-diethylamino-4-methyl-3-coumarinyl)phenyl]maleimide (CPM) Assay Buffer (20 mM HEPES, pH 7.5, 50 mM NaCl, 2 mM DDM, unless stated otherwise) prior to use. An amount of 5 mg/ml CPM stocks prepared in DMSO were stored at −80°C prior to use. CPM stocks were thawed at room temperature immediately before the experiment and diluted 25-fold into the appropriate assay buffer for use with DDM-solubilised protein or diluted 10-fold for SMA-solubilised protein. Substrate stocks were made at 60 mM in ddH_2_O, ethanol, or DMSO, with ddH_2_O as preference, depending on the requirement of the compound. Aqueous stocks were adjusted to pH 7.5 using either concentrated KOH or HCl before being aliquoted and stored at −80°C until use. For non-neutral substrates in non-aqueous stock solutions the concentration of HEPES in the final reaction mixture was increased to prevent a change in pH and neutral pH confirmed before testing. In thin walled optical cap PCR tubes (Bio-Rad), 2-8 µg of protein was diluted into 45 µl of CPM Assay Buffer containing substrates of interest and incubated for 10 min on ice before the addition of 5 µl appropriately diluted CPM. The reaction mixture was incubated for a further 10 min on ice with occasional mixing before transfer to a qPCR thermocycler (Thermo Fisher QuantStudio 3). The thermocycler programme was set to initially cool to 5°C at a rate of 1.6°C per second, before beginning a melt curve programme which ramped from 5°C to 95°C at a rate of 1.6°C/s, pausing for 5 s every 1°C to take fluorescence readings. Sybr Green fluorescence filters were used, with an excitation of 470 ± 15 nm and emission of 520 ± 15 nm. Data was exported for analysis in Microsoft Excel, and the trough of the derivative taken as *T*_m_. Temperature change was calculated against a baseline of zero substrate, with the addition of DMSO or ethanol, where necessary depending on the requirements of the substrate. Statistical significance was determined by a *t*-test comparison of *T*_m_ values observed in the presence and absence of compound.

### GFP-based thermostability (GFP-TS) assay

The GFP-TS thermostability assay was performed similarly to [43]. VcINDY-GFP fusions were expressed as described above. VcINDY-GFP-containing membrane vesicles were solubilised with DDM as described previously, aliquoted by volume, and stored at −80°C. For use in the GFP-TS assay, DDM-solubilised membrane samples were thawed and diluted with GFP-TS Reaction Buffer (20 mM Tris HCl, pH 7.5, 150 mM NaCl, 2 mM DDM, 3.5 mM β-OG) to a final dilution of 80 ml per litre of original culture. Chloroform solubilised lipid stocks (Avanti) were dried under a N_2_ stream, washed through solubilisation in pentane and dried again under the N_2_ stream, before solubilisation in GFP-TS Reaction buffer to a final concentration of 20 mg/ml.

For experiments examining the aggregation of VcINDY over a range of temperatures, 150 µl samples containing protein and substrates and/or lipids were heated for 10 min at 4, 20, 30, 40, 50, 60, 70, 80, 90, and 100°C before centrifugation at 18 000×***g*** at 4°C for 30 min. Supernatants and pellets (resuspended in 150 µl GFP-TS buffer) were transferred to a 96 well plate and GFP fluorescence was measured using a fluorescence plate reader (either a BMG LABTECH FLUOstar or a BMG LABTECH CLARIOstar). To avoid issues stemming from the reduction in GFP fluorescence caused by thermal denaturation of the GFP tag itself, the ratio of the fluorescence values between the supernatant and pellet was compared. *T*_m_ values were calculated by fitting each replicate to a sigmoidal dose response curve in R using the nls () function. For experiments examining the level of VcINDY aggregation occurring at a fixed temperature, samples were incubated in the absence or presence of 5 mg/ml of each lipid or lipid mixture at a temperature corresponding to the calculated *T*_m_ +5°C for 10 min, and then processed in the same manner as temperature range experiments. The fluorescence of the supernatant was divided by the fluorescence of the pellet in order to give Relative Stability, such that equal distribution is equal to 1, a higher proportion of soluble VcINDY is greater than 1, and a lower proportion of soluble VcINDY is less than 1. Relative stability changes were calculated as the difference between relative stability in the presence and absence of substrate. Significance was determined by a *t*-test comparison of ΔRelative Stability values in the presence of no lipids and in the presence of the lipids of interest.

## Results

### Experimental approach for ligand-induced thermostability measurements

**Figure 1.**
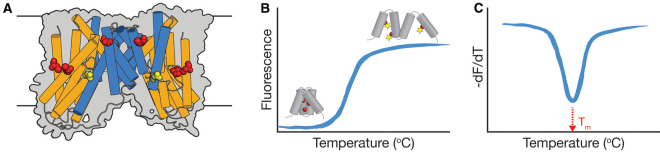
Principles of the thermal shift assay approach. (**A**) Cartoon representation of dimeric VcINDY showing the location of the three native cysteines (red spheres). The transport domain (orange cylinders), the scaffold domain (blue cylinders) and the substrate, succinate, in the inward-facing state substrate-binding site (yellow spheres) are indicated. (**B**) Cartoon representation of the unfolding curve of a protein (grey cylinders) depicting the temperature-dependent increase in fluorescence as the buried cysteines (red spheres) are exposed to the thiol-reactive fluorophore CPM (yellow stars). (**C**) Derivative of the unfolding curve (−dF/dT) with the apparent protein *T*_m_.

When proteins bind to specific ligands, be they substrates or inhibitors of the protein, the number of bonds formed increases, which leads to an increase in the overall stability of the protein. Ligand-induced stabilisation has been observed for several proteins, including transporters and globular proteins, and manifests itself as an increase in the melting temperature (*T*_m_) of the protein, which is measurable by several methods, including circular dichroism (CD), differential scanning fluorimetry (DSF), and radioactive ligand binding, among many others [44–46]. Here, we have employed a recently developed high-throughput fluorescence-based thermal shift assay to monitor VcINDY's propensity to bind to a wide variety of anionic compounds. In this thermal shift assay, detergent-solubilised and purified VcINDY, which has three native cysteine residues (Figure 1A), is incubated with a thiol-specific fluorophore, CPM, which is weakly fluorescent until conjugated to a cysteine [47, 48]. The protein:dye mixture is heated to thermally denature the protein, expose the buried cysteines to the solvent, and allow them to react with the CPM dye, which increases its fluorescence output (Figure 1B). A qPCR thermocycler is used to both monitor the fluorescence of the dye and thermally denature VcINDY, which is achieved by ramping the temperature from 20–95°C, producing a characteristic melting transition or melt curve (Figure 1B). VcINDY's *T*_m_ under a given set of conditions is the temperature at which the highest rate of unfolding is observed (Figure 1C), and ligand binding is deemed to have occurred if this observed *T*_m_ is modulated by the presence of the ligand.

**Figure 2.**
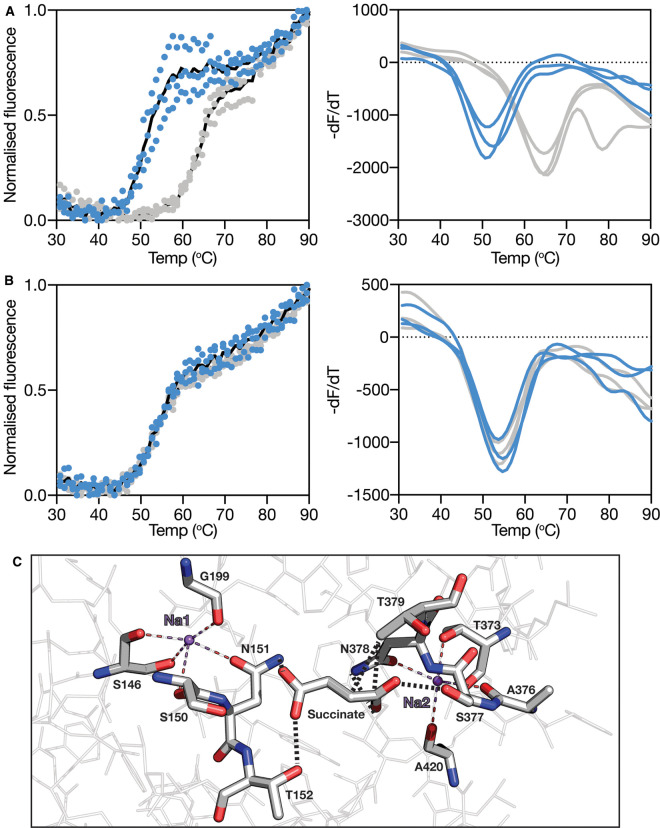
Substrate-induced thermostabilisation of VcINDY. Typical melt curve (left panel) and its derivative (right panel) of (**A**) VcINDY^WT^ and (**B**) VcINDY^SNT1AAA^ mutant in the absence (blue data) and presence (grey data) of 5 mM succinate (in the presence of 50 mM NaCl). Three data sets for each protein under each condition are shown. Black lines in the melt curves are the average of the three datasets shown. (**C**) Schematic of the succinate binding site of VcINDY and the two identified Na^+^ binding sites. Succinate and the two bound Na^+^ ions (Na1 and Na2, purple) are labelled, the amino acid residues involved in key substrate interactions are depicted, and the dashed lines indicate the interactions between the residues and the substrates.

To determine VcINDY's compatibility with this approach, we performed a basic temperature titration experiment in which we heated VcINDY from 20–95°C in the presence and absence of succinate, a model substrate for this transporter (Figure 2). In the absence of succinate (but in the presence of 50 mM NaCl), we observed a substantial temperature-dependent increase in fluorescence of the VcINDY:CPM mixture (Figure 2A, blue data); revealing VcINDY's *T*_m_ to be 51.7°C under these conditions. Upon addition of 5 mM succinate to the same reaction, we observed a substantial rightward shift of the melt curve (Figure 2A, grey data), and a significant increase in the *T*_m_ to 64.9°C, suggesting that this methodology is capable of reporting on VcINDY's substrate interactions.

To rule out the possibility that VcINDY's substantial succinate-induced stabilisation is due to non-specific interaction with succinate, we repeated the assay with a mutant of VcINDY with three critical substrate binding residues mutated to alanine, namely, S149, N150, and T151, from the tip of re-entrant loop, HP1 (Figure 2C). This mutant, VcINDY^SNT1AAA^ is stable, as demonstrated by the monodisperse size exclusion chromatography trace (Supplementary Figure S1A), but is incapable of catalysing Na^+^-driven transport of succinate (Supplementary Figure S1B), presumably due to an inability to bind succinate. Applying the same temperature ramp to VcINDY^SNT1AAA^ as for VcINDY^WT^, we observed that in the absence of succinate both wildtype and mutant had very similar *T*_m_s, with VcINDY^SNT1AAA^ having a slightly elevated *T*_m_ of 54.0°C, demonstrating that the binding site mutants did not compromise the structural integrity of the protein (Figure 2B, blue data). However, unlike VcINDY^WT^, we observed no succinate-dependent increase in the *T*_m_ of VcINDY^SNT1AAA^ (Figure 2B, grey data), demonstrating that the succinate-induced stabilisation of VcINDY is due to specific binding site interactions between protein and ligand.

### Probing VcINDY's cation interactions

While it is well established that a Na^+^ gradient, and to a considerably lesser extent a Li^+^ gradient, can power transport of succinate by VcINDY [8], the effect of cations on succinate *binding*, rather than the full transport cycle, has not been explored for any DASS transporter. Using our newly established thermal shift assay, we sought to explore the relationship between the identity and concentration of cation and the stability of VcINDY in general, and how the cation conditions influence succinate-induced stabilisation.

**Figure 3.**
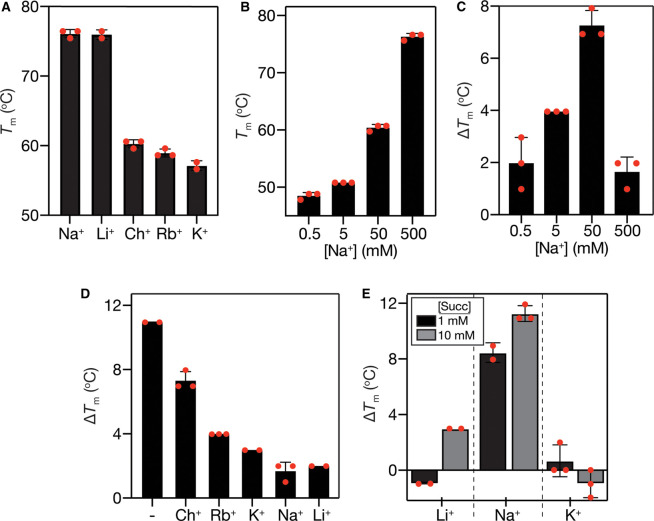
Cation dependence of VcINDY's interactions with succinate. (**A**) Stabilising effect of a range of cations on VcINDY. An amount of 500 mM of each cation, as their Cl salts, was added to each reaction. (**B**) Dose response of Na^+^ on the stability of VcINDY. The ionic strength of the reaction was balanced with choline chloride. (**C**) Dose response of Na^+^ on the succinate-induced stabilisation (Δ*T*_m_) of VcINDY. The ionic strength of the reaction was balanced with choline chloride. (**D**) Succinate-induced stabilisation of VcINDY in 50 mM Na^+^ in the absence (−) or presence of an additional 450 mM cation. (**E**) Succinate-induced stabilisation of VcINDY in the presence of 50 mM Li^+^, Na^+^ or K^+^ with a final succinate concentration of 1 or 10 mM. Individual datapoints are shown as red circles and the error bars represent SD. Raw derivative plots of unfolding curves related to this data can be found in Supplementary Figure S2.

We first wished to determine whether the presence of Na^+^ could stabilise VcINDY over and above the stabilisation effects of other cations. To do this, we determined the *T*_m_ of VcINDY in the presence of Na^+^ and compared it to the stabilisation imparted by a range of cations that are either unable to support succinate transport by VcINDY (K^+^ and choline^+^), or catalyse transport poorly (Li^+^) [8]. In the presence of 500 mM Na^+^, VcINDY's *T*_m_ is 76°C, which is identical with the stabilisation imparted by the presence of Li^+^ and considerably higher than the stabilisation resulting from K^+^, Rb^+^ and Choline^+^ at the same concentration (Figure 3A and Supplementary Figure S2A). These data suggest that Na^+^ specifically stabilises VcINDY, which is consistent with it acting as a Na^+^-driven symporter [8]. However, it is interesting to note that Li^+^ stabilises VcINDY to almost exactly the same extent (*T*_m_ in Li^+^ was 76.0 ± 0.5°C, and 76.2 ± 0.5°C in Na^+^), implying that the same number of bonds are formed, and suggesting that the vast difference between Na^+^- and Li^+^-driven transport rates is not due to VcINDY's inability to bind Li^+^ properly. We observed that Na^+^-induced stabilisation of VcINDY was dose-dependent with its *T*_m_ increasing from 48°C to 76°C between 0.5 and 500 mM Na^+^ (Figure 3B and Supplementary Figure S2B).

We next explored the dose dependence of Na^+^ on the succinate-induced stabilisation of VcINDY. As the binding of coupling ions is often directly coupled to substrate binding [49], we hypothesised that we would see an increase in the stabilising effects of succinate as we increased the Na^+^ concentration. Indeed, we observed substantial increases in the succinate-induced stabilisation as we increased the Na^+^ concentration from 0.5 mM to 50 mM (ionically balancing with choline), which is approximately the *K*_M_ for Na^+^ (Figure 3C and Supplementary Figure S2B) [8]. These data suggest that Na^+^ binding is required for the priming of the succinate binding site, as has been seen in other elevator-like transporters [50, 51]. Interestingly, increasing the Na^+^ concentration to 500 mM abolished succinate-induced stabilisation (Figure 3C and Supplementary Figure S2B). We reasoned that the high ionic strength of the reaction buffer may interfere with VcINDY's ability to bind the dianionic succinate. To test this, we performed a ‘cation interference’ experiment wherein we monitored the stabilising effects of succinate on VcINDY in the presence of 50 mM Na^+^ alone or in the presence of 50 mM Na^+^ in addition to 450 mM choline^+^, Rb^+^, K^+^, Na^+^ or Li^+^. If the weak succinate-induced stabilisation is due simply to high ionic strength, we would expect to see a similar affect in the presence of *any* cation. Indeed, succinate-induced stabilisation was substantially reduced in the presence of high cation concentrations, regardless of the cation (Figure 3D and Supplementary Figure S2C). These data indicate that a charge dependent component of the substrate-binding site (potentially the bound Na^+^ ions or helical dipoles) is shielded by the high ionic strength buffer, suggesting a charge-based aspect of this ordered binding model. Consistent with this model, each cation did not display equal inhibitory properties: cations with the smaller ionic radii (Li^+^ and Na^+^) were able to inhibit succinate binding to greater degree than slightly larger ions (Rb^+^ and K^+^), which inhibited more efficaciously than still larger ions (choline^+^), suggesting that ready access to the binding site is required for inhibition.

These data illustrate Li^+^ and Na^+^ can stabilise VcINDY to the same degree (Figure 3A), clearly implying that these cations interact with VcINDY in an identical fashion with the same number of bonds being formed. We reasoned that the inability of a Li^+^ gradient to support robust succinate transport rates (compared with Na^+^) may be due to altered succinate binding in the presence of Li^+^ compared with Na^+^ [8]. We tested this possibility by monitoring succinate-induced stabilisation in the presence of 50 mM Li^+^, Na^+^ and K^+^ (Figure 3E). In the presence of Na^+^, the presence of 1 mM succinate stabilised VcINDY by a substantial 8°C, whereas, the same succinate concentration induced negligible stabilisation in the presence of Li^+^ (Figure 3E and Supplementary Figure S2D). Only when succinate concentration was increased to 10 mM was a moderate succinate-induced stabilisation in the presence of Li^+^ observed. K^+^, which does not support succinate transport by VcINDY [8], did not facilitate any succinate-induced stabilisation at either high or low succinate concentration (Figure 3E and Supplementary Figure S2D). Taken together, these data suggest that, although Li^+^ is able to bind to VcINDY similarly to Na^+^ while carrying the same charge, Na^+^ binding primes the succinate binding site through local allosteric changes or direct contacts with the substrate that cannot be replicated by Li^+^.

### Large-scale compound screen reveals VcINDY's ligand requirements and unearths novel interacting compounds

**Figure 4.**
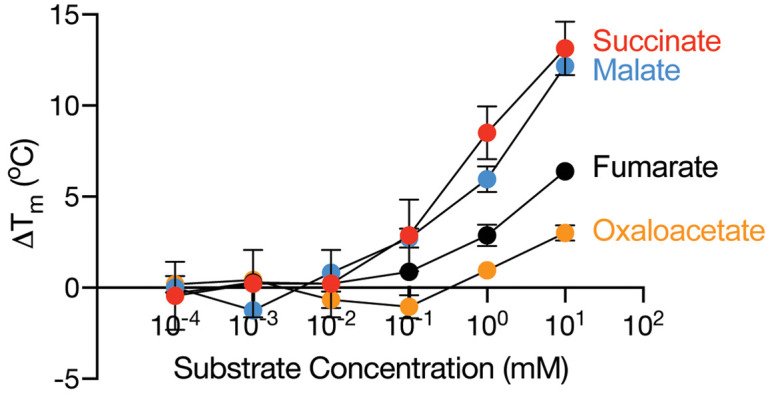
The effect of substrate concentration on the *T*_m_ of VcINDY. Change in melting temperature (Δ*T*_m_) of VcINDY as a function of substrate concentration. The substrates tested are succinate (red data), malate (blue data), fumarate (black data) and oxaloacetate (orange data). Data presented are the average of triplicate data sets and the error bars represent standard deviation. Representative raw derivative plots of VcINDY unfolding under these conditions are presented in Supplementary Figure S3.

A previous study using a succinate transport competition assay identified that VcINDY interacts with several divalent anions, including several C_4_-dicarboxylates [8]. To further expand on the repertoire of compounds that can interact with VcINDY, as well as define some of the ‘rules’ for substrate interactions, we tested a library of 71 compounds, focusing primarily on anions, for their propensity to interact with VcINDY. However, before undertaking this task, we first determined the appropriate concentration of substrate to use in the assay by monitoring the change in *T*_m_ in the presence of increasing concentrations of the known VcINDY substrates, succinate, malate, fumarate, and oxaloacetate (Figure 4 and Supplementary Figure S3).

Using the CPM-based thermal shift assay, we observed dose-dependent enhancement in the thermostability of VcINDY in the presence of each of the known substrates, with higher substrate concentrations leading to increased thermostabilisation (Figure 4 and Supplementary Figure S3). As is the case for other transporters probed using CPM-based thermal shift assays [47], we only observed substantial stabilisation at substrate concentrations considerably higher than the *K*_M_ for transport; VcINDY's Km for succinate is 1 µM [8], whereas substantial stabilisation by succinate was only observed at >100 µM (Figure 4). Based on these data, to obtain the greatest signal:noise ratio in our substrate screen, we selected 10 mM as the final concentration of each member of compound library.

Of the 71 compounds tested using this thermal shift assay, 18 were identified that significantly increased the stability of VcINDY (Supplementary Figure S4). For clarity, we have divided the large interaction screen into smaller groups of compounds: linear dicarboxylates of varying chain length, modified C_4_-dicarboxylates, modified C_5_-dicarboxylates, aromatic dicarboxylates, and long branched carboxylates. Where potential ligands were not sufficiently soluble in aqueous solution, either ethanol or DMSO were used as the compound solvent. In these cases, to determine the level of stabilisation, we compared VcINDY's *T*_m_ in the presence of the compound to the *T*_m_ in the presence of the solvent alone; DMSO alone has a slight stabilising effect (Supplementary Figure S5B, whereas ethanol alone destabilises VcINDY (Supplementary Figure S5C)

#### The chain length of dicarboxylates determines its ability to bind

**Figure 5.**
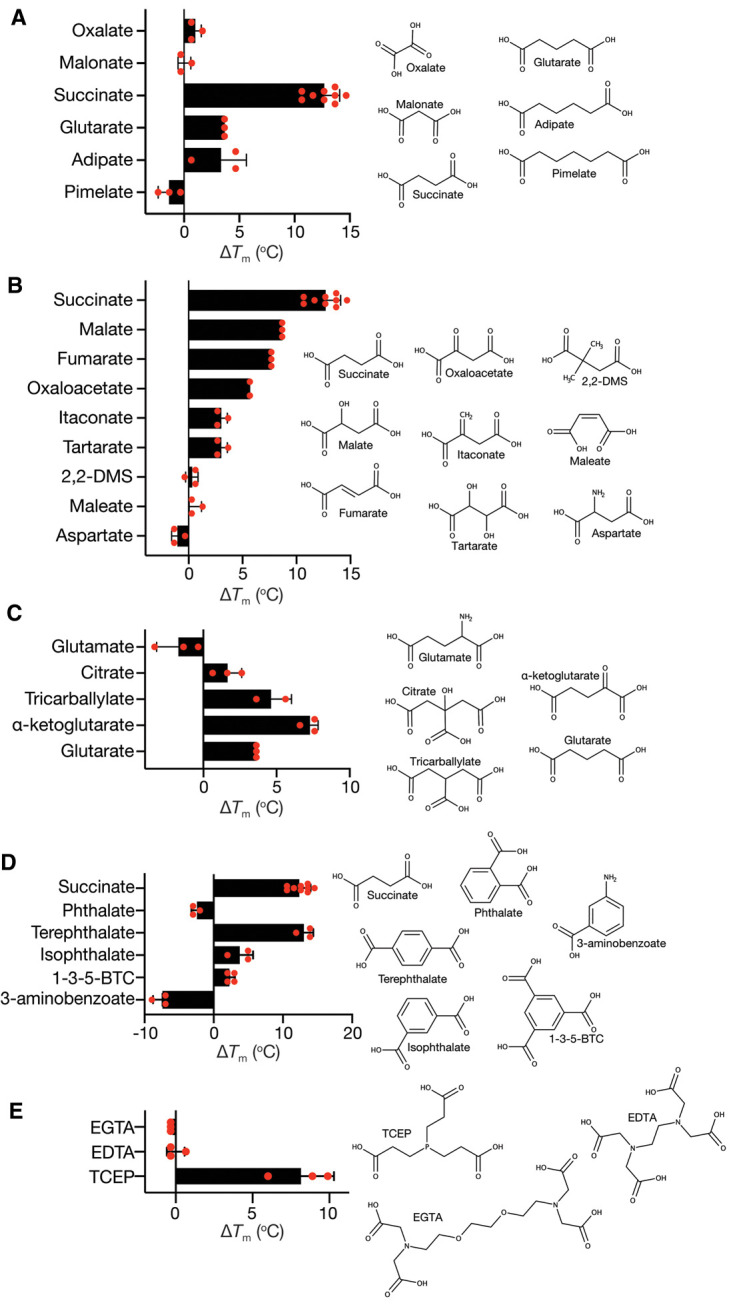
Thermostability screen in the presence of selected groups of anions. The melting temperature shift (Δ*T*_m_) of VcINDY in the presence of each compound was determined by subtracting the VcINDY *T*_m_ in the absence of substrate (in the presence of 50 mM Na^+^) from the *T*_m_ in the presence of Na^+^ and the indicated compound. The compounds selected for comparison are; (**A**) dicarboxylates with increasing chain lengths; (**B**) C_4_-dicarboxylate modifications; (**C**) C_5_-dicarboxylate modifications; (**D**) aromatic dicarboxylates; and (**E**) long branched carboxylates. The average value from at least duplicate datasets is shown, individual datapoints are shown as red circles and error bars represent SD. Chemical structures of each compound is shown. The full compound screen is shown in Supplementary Figure S4. 2,2-DMS, 2,2-dimethylsuccinate; 1,3,5-BTC, 1,3,5-benzene tricarboxylate; EGTA, ethylene glycol-bis (2-aminoethylether)-*N*,*N*,*N′*,*N′*-tetraacetic acid; EDTA, ethylenediaminetetraacetic acid; TCEP, tris (2-carboxyethyl)phosphine.

As previously observed using transport competition assays, dicarboxylate binding to VcINDY is highly dependent on the chain length, with the four carbon succinate providing maximal stabilisation (Figure 5A and Supplementary Figure S5A). We also observed modest stabilisation by glutarate (C5) and adipate (C6), but not pimelate (C7), which is consistent with previous competition assay data, which revealed modest inhibition by glutarate and adipate, but no inhibition from pimelate [8].

The crystal structures of VcINDY have revealed that the side chain amide of N151 and side-chain hydroxyl of T152 form hydrogen bonds with one of the carboxylate groups of succinate, while the side-chain hydroxyl of S377 and side-chain amide of N378 interact with the other carboxylate group (Figure 2C) [12, 13]. Given that none of the monocarboxylates tested, including, carbonate, gluconate, glyoxylate, acetate, formate, bicine, tricine, creatine, and all amino acids (except for alanine, which modestly, but significantly, destabilised VcINDY), had any effect on the stability of VcINDY, it is likely that both of the carboxylate binding sites must be satisfied for efficacious binding. Therefore, while unsaturated dicarboxylates with 4–6 carbons can enter the binding site and adopt a suitable conformation, smaller dicarboxylates cannot bridge the gap between the carboxylate binding sites, and longer dicarboxylates cannot orient within the binding site to accommodate both binding sites. In this way, VcINDY is able to generate substrate specificity, in part, by implementing a molecular length selectivity filter.

#### C_4_-dicarboxylate modifications

As the C_4_-dicarboxylates appear to be VcINDY's favoured ligands, we investigated how modifications of the model substrate, succinate, affects its stabilising ability. Both inward-facing state structures of VcINDY reveal that succinate is bound in an extended conformation [12, 13], reinforcing the suggestion that a major substrate selection criterion is the length of the dicarboxylate. This requirement for an elongated posture is further supported by the observation that fumarate, which has an enforced extended conformation due to the C = C between the second and third carbons, imparts 8°C stabilisation to VcINDY. In contrast, the *cis* isomer, maleate, which has an enforced contracted posture has no stabilising ability (Figure 5B and Supplementary Figure S5A)). Discrimination between fumarate and maleate has been shown previously for VcINDY and other members of the DASS family, suggesting that the elongated ligand posture is a conserved requirement [8, 25, 52, 53].

The addition of a hydroxyl or ketone group to the C2 of succinate, to make malate and oxaloacetate, respectively, was tolerated and resulted in substantial stabilisation of VcINDY, albeit lower than succinate (Figure 5B and Supplementary Figure S5A). These C2 functional groups are likely accommodated similarly to citrate, wherein the additional polar groups are oriented towards the cytoplasmic opening of the binding site where they could interact favourably with water [12, 13]. However, unlike citrate, both malate and oxaloacetate are transportable substrates of VcINDY [8]. Itaconate, which has a C2 methylene group exhibits reduced stabilisation compared with malate and fumarate, possibly due to the lack of hydrogen bonding potential of this functional group. Tartarate, a C_4_-dicarboxylate with hydroxyls on both carbons 2 and 3, stabilises VcINDY, but to a lesser extent than malate, which only has the C2 hydroxyl, for reasons as yet unknown. Aspartate, which has a C2 amine group, is unable to stabilise VcINDY, which is likely due to repulsion of this extra positive charge from the already positively charged binding site that has at least 2 vicinal Na^+^ ions and positive helical dipoles [13]. Finally, 2,2-dimethlysuccinate (2,2-DMS), which has two additional methyl groups does not stabilise VcINDY, likely due to steric hindrance (Figure 5B and Supplementary Figure S5A).

#### C_5_-dicarboxylate modifications

As the C_5_-dicarboxylate, glutarate displayed appreciable stabilisation of VcINDY (Figure 5C and Supplementary Figure S5A), we sought to determine how modifications of this backbone affect interaction with VcINDY. The binding of citrate, which is essentially a C_5_-dicarboxylate with a hydroxyl and a carboxyl group on carbon 3, has been well characterised and is present in the crystal structures of VcINDY from both structural studies [12, 13]. However, we have previously shown that citrate is a very low affinity inhibitor of VcINDY and is not a transported substrate [8]. The citrate-bound structures of VcINDY reveal that citrate is accommodated by the substrate-binding site by projecting the hydroxyl and carboxylate groups into the cytoplasmic opening of the binding site, where these functional groups make no contacts with the protein [12, 13]. In our assay, the addition of 10 mM citrate resulted in a modest stabilisation of VcINDY, which is not surprising given that inhibition of succinate transport was not observed with less than 50 mM citrate [8]. Interestingly, we observed substantially greater stabilisation by tricarballylate, which is structurally very similar to citrate, but without the hydroxyl group on C3 (Figure 5C and Supplementary Figure S5A). In the absence of structural information, it is not clear what difference this small change makes to the protein:ligand interactions. Amidation of C4 of glutarate to make glutamate renders the molecule incapable of interacting with VcINDY (Figure 5C and Supplementary Figure S5A); similarly to aspartate, it is likely that the introduction of positive charge to the ligand leads to electrostatic repulsion from the binding site. Finally, α-ketoglutarate stabilises VcINDY to a greater extent than glutarate, which conforms with the observation that α-ketoglutarate appreciably inhibited succinate transport in the previous transport competition assay [8].

#### Aromatic dicarboxylates

To probe the flexibility of VcINDY's substrate-binding site, we tested a range of phthalic acid isomers; terephthalate (para-isomer), isophthalate (meta-isomer) and phthalate (ortho-isomer), which have two carboxylate groups in different positions on a planar benzene ring (Figure 5D). While both tere- and isophthalate substantially stabilised VcINDY, suggesting some degree of flexibility in the binding site, phthalate failed to interact with VcINDY at all (Figure 5D and Supplementary Figure S5C). Similarly to maleate, phthalate's inability to interact with VcINDY is likely due to its failure to contact both carboxylate binding regions in VcINDY's binding site. The addition of a third carboxylate group to isophthalate to make 1,3,5-benzentricarboxylate (1,3,5-BTC) modestly increased its stabilising properties (Δ*T*_m_ of 2.5°C, Figure 5D and Supplementary Figure S5C). In the cases of aspartate and 3-aminoglutarate, the presence of an amine group, which imparts a positive charge to the compound at physiological pH, prevents interactions with VcINDY. However, amidation of a benzene monocarboxylate to form 3-aminobenzoate leads to significant destabilisation of VcINDY by a substantial 7.6°C (Figure 5D and Supplementary Figure S5B).

#### Long-branched carboxylates

**Figure 6.**
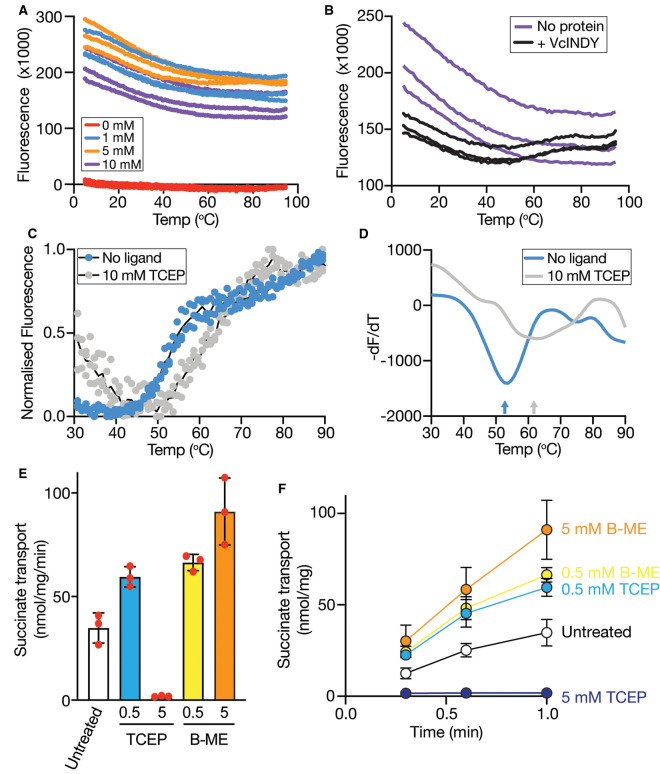
TCEP directly interacts with VcINDY and can inhibit transport activity. (**A**) CPM-derived fluorescence in the presence of 0 mM (red data), 1 mM (blue), 5 mM (orange) or 10 mM (purple) TCEP. Three replicates for each concentration are shown. (**B**) CPM-derived fluorescence in the presence of 10 mM TCEP with (black data) and without (purple) VcINDY present. (**C**) Melt curve of VcINDY in the absence (blue data) and presence of (grey data) 10 mM TCEP. Three data sets for each condition are shown and black lines represent the average of the datasets shown. (**D**) Representative derivative plot for VcNDY in the presence (grey data) and absence (blue data) of 10 mM TCEP. Colour coded arrows indicate the *T*_m_ under each condition. (**E**) Na^+^-driven [^3^H]-succinate transport activity of VcINDYcysless after purification and reconstitution into proteoliposomes in the absence of reducing agent (untreated) or the presence of 0.5 mM TCEP, or 5 mM TCEP, 0.5 mM β-mercaptoethanol (B-ME), or 5 mM BME. (**F**) Transport of [^3^H]-succinate over time under the same conditions as panel (**C**). The 1 min timepoint is plotted in panel (**C**). Data are an average of a triplicate dataset and the error bars represent SD. Error bars not visible are smaller than the data point symbol.

Having observed stabilising effects of the tricarboxylates citrate and tricarballylate as well as flexibility in the length of dicarboxylate that is recognised, we wished to determine the effects of increasing the branch length of tricarboxylates. To test this, we probed the stabilising ability of the chelating agents EDTA and EGTA, but they had no effect on VcINDY stability (Figure 5E and Supplementary Figure S5A). The other long branched carboxylate tested was the reducing agent tris (2-carboxyethyl)phosphine (TCEP) (Figure 5E), which not only stabilises VcINDY, but did so substantially better than citrate and tricarballylate. TCEP dramatically increases CPM-based fluorescence and is considered incompatible with this CPM-based Thermofluor approach [54]. To determine how TCEP affects our assay, we monitored CPM fluorescence in the absence of protein but in the presence of increasing TCEP concentrations (Figure 6A). Addition of even 1 mM TCEP to the reaction resulted in a substantial increase in background fluorescence that decreased non-linearly as a function of temperature (Figure 6A). Interestingly, the level of background fluorescence was not TCEP concentration-dependent; in fact, the lowest background observed was in the presence of 10 mM TCEP (Figure 6A). Despite this high fluorescence background in the presence of 10 mM TCEP, we were able to observe a clear melt curve for VcINDY under these conditions by simply increasing the amount of the protein from the standard 2 µg we used previously to 8 µg (Figure 6B, black data). By comparing the *T*_m_ of VcINDY in the presence and absence of 10 mM TCEP (Figure 6C,D), we were able to calculate a substantial Δ*T*_m_ of 8.3°C, suggesting that TCEP can interact with VcINDY.

Due to the unexpected nature of this finding and the known complications of including TCEP in this CPM-based thermofluor assay, we employed a complementary approach to evaluate whether TCEP can interact with VcINDY. In this approach, we monitored transport of [^3^H]-succinate into VcINDY-containing liposomes in the presence of 0.5 mM and 5 mM TCEP (Figure 6E,F); if TCEP can interact directly with VcINDY one would expect inhibition of succinate transport. In the presence of 0.5 mM TCEP we observed a slight increase in activity compared with transport in the absence of TCEP (Figure 6E,F). However, increasing the TCEP concentration to 5 mM completely abolished succinate transport (Figure 6E,F). To control for unwanted effects of high reducing agent concentration on the two native cysteines of VcINDY, we performed this assay with cysteine-free VcINDY, which is capable of robust Na^+^-driven transport [8]. Furthermore, to rule out the possibility that the high concentration of reducing agent had an unforeseen effect on the lipids that could inhibit transport, we also performed this competition assay in the presence of β-mercaptoethanol (B-ME, Figure 6E,F). Rather than inhibiting transport, we observed an increase in transport activity as a function of B-ME concentration (Figure 6E,F). Taken together, these data demonstrate that TCEP is able to form direct interactions with VcINDY that renders it incapable of transport, and given the considerably different properties of TCEP to other known binders, it is possible that TCEP has a notably different mode of binding. We note with interest that the recent crystal structure of VcINDY was solved in the presence of TCEP, although no density for the molecule was reported [13].

To investigate whether the compounds found to stabilise VcINDY^WT^ are interacting with the known binding site or whether they are binding in an alternative region of the protein, we assessed whether the binding site mutant VcINDY^SNT1AAA^ could be stabilised by the compounds that most efficaciously stabilised VcINDY^WT^’; we tested terephthalate, succinate, fumarate, malate, a-ketoglutarate, oxaloacetate, tricarballylate and TCEP (Supplementary Figure S6). All of the compounds tested were able to modestly stabilise VcINDY^SNT1AAA^, suggesting that the mutant retains the ability to bind these compounds at this concentration (10 mM, (Supplementary Figure S6)). Importantly though, the level of stabilisation of VcINDY^SNT1AAA^ by each compound was substantially lower than the extent to which they stabilised VcINDY^WT^, suggesting that the three binding site residues (S150, N151 and T152, Figure 2C) were involved with these interactions, and that these compounds interact with the known binding site of VcINDY.

Taken together, data from our large-scale interaction screen suggests that VcINDY's binding site can tolerate numerous modifications to the succinate backbone as long as 1) both carboxyl groups can reach their respective binding sites, 2) any additional functional groups can orient towards the binding site opening, and 3) no additional positive charges are introduced. In addition, we have identified several new families of compounds that interact with VcINDY; the structural basis of which may prove important for the development of future DASS transporter inhibitors.

### Differential effects of lipids on protein stabilisation suggest complex interplay between substrate and lipid interactions in VcINDY

**Figure 7.**
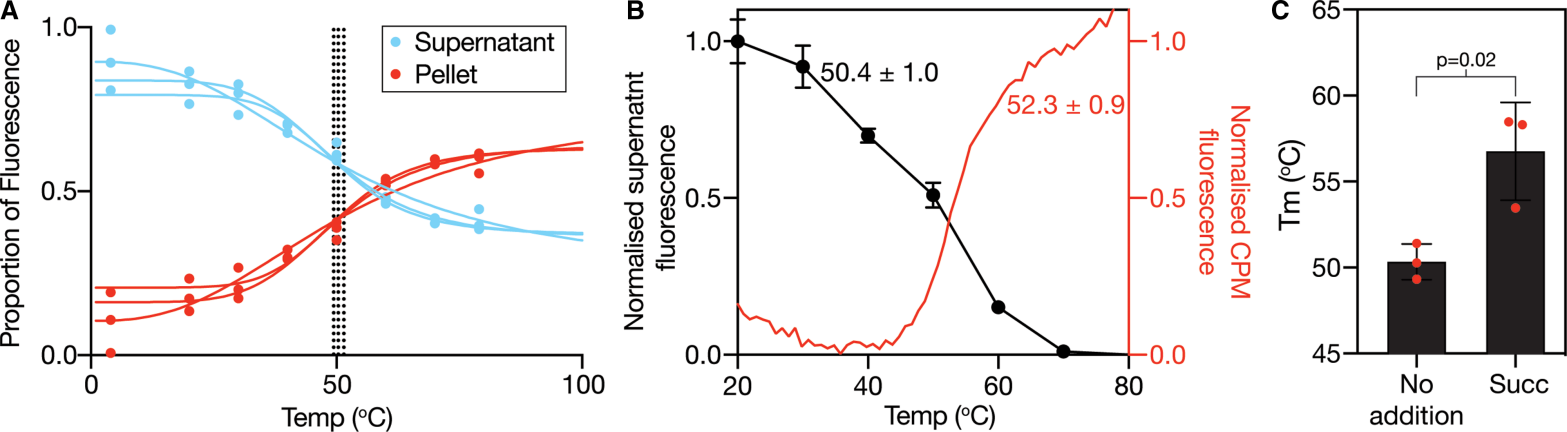
Validation of the GFP-TS approach with detergent-solubilised VcINDY. (**A**) Proportion of the GFP fluorescence in the supernatant (blue data) and pellet (red data) as a function of incubation temperature. (**B**) Comparison of VcINDY's *T*_m_ derived from the GFP-TS approach (black data) and the CPM-based thermal shift assay using normalised data from each method. GFP-TS data are the average of triplicate data sets and the error bars represent SEM. (**C**) *T*_m_ of VcINDY in the presence and absence of 5 mM succinate. Results are the average of triplicate data sets, single datapoints are shown as red circles, the error bars represent SD, and significance was determined using a two-sided *t*-test.

As we were able to use the thermal shift assay to discriminate between binders and non-binders of detergent-solubilised VcINDY, we wished to examine whether the observed substrate-induced stabilisation could be reproduced in a lipid environment, and what effect the lipid might have on these interactions. To initially examine the effects of lipids on succinate interactions with detergent-solubilised VcINDY we titrated lipids into the reaction mixture of the standard CPM-based thermal shift assay (as used in Figure 2). However, the inclusion of lipids in the reaction resulted in direct lipid:dye interactions, which generated a high background fluorescence signal that obscured VcINDY's melt curve, making analysis impossible at anything other than very low lipid concentrations (Supplementary Figure S7). Instead, we turned to a recently developed aggregation-based thermal shift assay (GFP-TS) shown to be highly compatible with screening protein:lipid interactions [43]. In this approach, GFP-tagged protein is heated in the presence of the detergent octyl-β-d-glucoside (β-OG), which encourages aggregation of denatured protein [43]. The heat-denatured proteins were sedimented by centrifugation and the distribution of GFP fluorescence between the supernatant and pellet is an accurate reporter on the level of protein denaturation at each temperature. To assess whether this approach would be compatible with VcINDY, we generated GFP-VcINDY fusion and determined its *T*_m_ using GFP-TS. Importantly, the GFP-VcINDY fusion was capable of robust Na^+^-driven succinate transport indicating that it will report faithfully on relevant substrate interactions (Supplementary Figure S8). We assessed VcINDY's *T*_m_ using GFP-TS by examining the ratio of the fluorescence present in the supernatant and pellet at different temperatures (Figure 7A). This revealed a *T*_m_ of 50.4 ± 1.0°C, which is consistent with the *T*_m_ obtained by our CPM-based approach under the same conditions (Figure 7B), thus validating this alternative approach and demonstrating that the GFP fusion was not inadvertently altering VcINDY's stability. To test whether we could also observe the stabilising effects of succinate on VcINDY using GFP-TS, we determined the *T*_m_ of GFP-VcINDY in the absence and presence of 5 mM succinate, which revealed a 7°C succinate-induced stabilisation (Figure 7C).

To determine the impact of lipids on the stability of VcINDY and its interactions with substrate, we measured the proportion of protein remaining in the supernatant after incubating the protein at the *T*_m_+5°C. The proportion of protein remaining in the supernatant under different conditions would reveal the relative stability of the protein, which is the variable of interest, and preclude the need for full melting curves, increasing the throughput of the process [43]. In this variant of the assay, a higher proportion of GFP-VcINDY remaining in the supernatant is indicative of an increase in stability under that condition.

**Figure 8.**
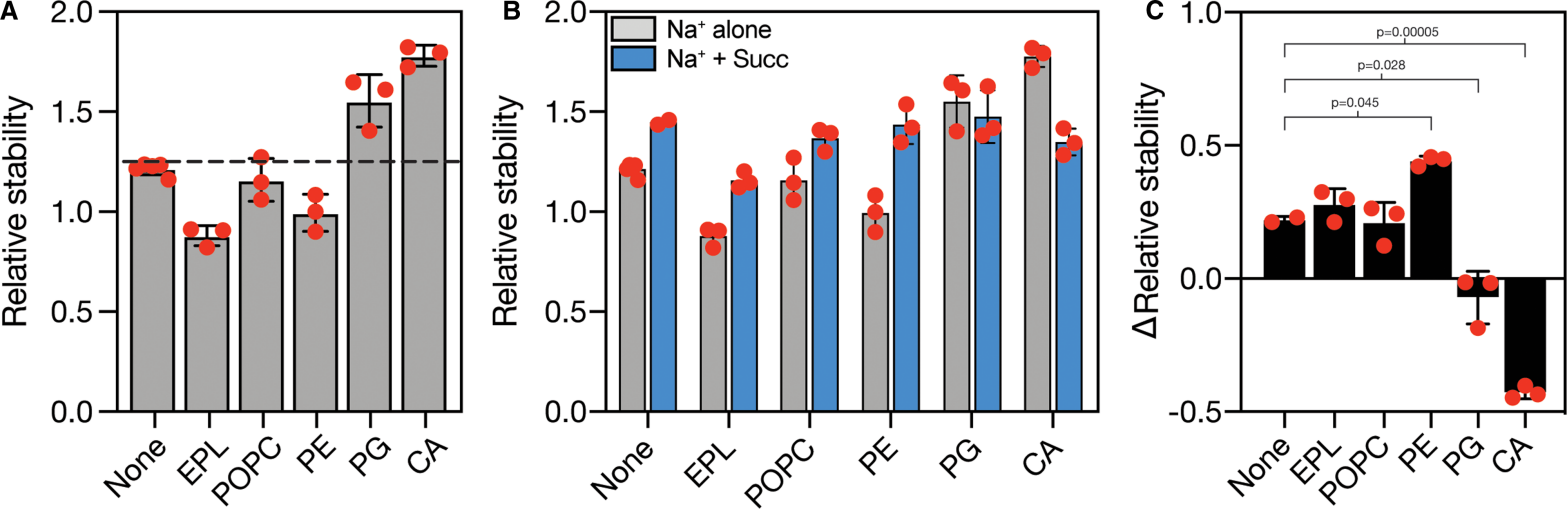
The effects of lipid headgroups on the stability of VcINDY in the presence and absence of succinate. (**A**) The relative stability (fluorescence in supernatant/fluorescence in pellet) of VcINDY in the absence of additional lipid (‘none’) or in the presence of *E. coli* polar lipid extract (EPL), 1-palmitoyl-2-oleoyl-glycero-3-phosphocholine (POPC), and purified *E. coli* phosphatidylethanolamine (PE), phosphatidylglycerol (PG) and cardiolipin (CA). Dashed line indicates relative stability of the no additional lipid control for comparison across the samples. (**B**) Relative stability of VcINDY in the presence of the same lipid panel ad in (**A**) in the presence (blue bars) and absence of succinate. The data from (**A**) is reproduced here (grey bars) for comparison. (**C**) The Δrelative stability (relative stability in the presence of succinate — the relative stability in the absence) of VcINDY in the same panel of lipids and lipid mixtures as in (**A**). Single datapoints are shown in red, error bars represent SD, and significance was determined using two-sided *t*-test.

We first assessed the effects of lipids on VcINDY stability in the absence of substrate. In this study, we have limited our investigation to comparing the effects of lipid headgroup on protein *T*_m_ and have not controlled for changes to the acyl chain length or saturation. To do this, we monitored the relative stability of GFP-VcINDY in Na^+^-containing buffer either in the absence of additional lipids, or presence of *E. coli* polar lipids extract (EPL), which is composed of ∼67% (w/w) phosphatidylethanolamine (PE), 23.2% phosphatidylglycerol (PG), and 9.8% cardiolipin (CA) (Avanti Polar Lipids). Compared with the stability in the absence of additional lipids, the addition of EPL significantly destabilised VcINDY (Figure 8A), presumably caused by a specific interaction with one or more lipids in the EPL mixture. To determine whether this destabilisation was specifically due to a component of EPL or caused by the mere presence of a lipid, we monitored the relative stability of VcINDY in the presence of the synthetic non-bacterial lipid POPC (1-palmitoyl-2-oleoyl-glycero-3-phosphocholine). Co-incubation with POPC resulted in no significant change in the stability of VcINDY indicating that POPC does not interact with the protein, and suggesting that the EPL-induced destabilisation was due to a specific interaction (Figure 8A). To investigate this in more detail and to determine which component of EPL induced VcINDY destabilisation, we assessed the effects of each component part of EPL in isolation by monitoring the relative stability of VcINDY in the presence of purified *E. coli* PE, PG or CA. Interestingly, co-incubation with PE destabilised VcINDY to a similar extent as EPL, whereas PG and CA substantially boosted stability, indicating that the three components of EPL differentially influence VcINDY's stability under these conditions (Figure 8A).

As these assays were performed in the presence of Na^+^ alone, we wished to determine how the lipid-induced stability effects were modulated by the addition of the model substrate, succinate (Figure 8B,C). Consistent with our previous data in detergent, in the presence of the control lipid, POPC, VcINDY was stabilised to same degree as in the absence of additional lipids. While EPL destabilised the protein in Na^+^ alone, the extra stabilisation caused by the addition of succinate was the same as in the absence of additional lipid (Figure 8B,C). However, in the presence of the purified *E. coli* PE, PG and CA we observed differential effects on succinate-induced stabilisation of VcINDY with the presence of PE leading to a substantially greater stabilisation than in the absence of additional lipid suggesting a synergistic effect of lipid and substrate; the presence of PG leading to no observable stability difference in the presence of succinate; and the presence of CA leading to substantial destabilisation upon addition of succinate (Figure 8B,C). These data combined suggest that the influence of added lipids on VcINDY is modulated by the substrate-bound condition of VcINDY, and may substantially impact the transport mechanism in the context of the complex lipid bilayer.

## Discussion

In this work, we have used a series of thermostability-based binding assays to reveal insight into the interactions of cations, substrates and lipids with VcINDY, which is the structural model for the DASS transporter family. While all previous mechanistic studies of DASS transporters have monitored transport, the results presented here marks the first time that substrate binding events have been studied in isolation. Using this approach, we have demonstrated that Na^+^ is able to bind to VcINDY in the absence of substrate, and that Na^+^ binding is a strict prerequisite for succinate interactions, revealing an ordered binding process. We also demonstrate that Li^+^, while carrying the same charge and stabilising VcINDY to the same extent as Na^+^, is unable to promote efficient succinate binding, suggesting that Na^+^-specific priming of the substrate-binding site is required for efficient transport. Using a large-scale screen of diverse anionic compounds, we have observed that substrate binding is largely governed by a molecular ruler-like selectivity filter wherein successful substrate interactions are dependent on the precise positioning of two carboxyl groups in the ligand. In addition, we have identified several new compounds that interact with VcINDY, which could form the basis of future DASS inhibitor design. Finally, we have demonstrated that there is a complex interplay between substrate and lipid interactions. While we can only speculate on the mechanistic implications of this interplay, our data hints that the substrate bound condition of the protein influences lipid interactions, or vice versa, which may have profound implications for the transport regulation in a complex lipid environment.

### Na^+^ and Li^+^ both interact with VcINDY, but only Na^+^ primes the protein for efficient succinate binding

Our data demonstrate that Na^+^ is able to bind to VcINDY in the absence of substrate (Figure 3A), and that the presence of Na^+^ is essential for substrate binding (Figure 3C), demonstrating an ordered binding model wherein at least one Na^+^ ion binds prior to substrate. While the requirement for the pre-binding of Na^+^ has been previously postulated and strongly supported by the recent structural analysis of the Na^+^-only bound state of VcINDY [4, 55], the actual effects of Na^+^ binding on succinate binding have not been explored. Structural studies have identified a dearth of positively charged amino acids in VcINDY's binding site leading to the suggestion that the binding of Na^+^ ions (in addition to helical dipoles) promotes anionic substrate binding via a charge-based attraction [13]. This suggestion is supported by our observation that high cation concentrations interfere with succinate binding, presumably by masking the binding site charge (Figure 3D). However, our data suggest that the effects of Na^+^ binding on substrate interactions go beyond mere electrostatic attraction. While previous work has demonstrated that a Li^+^ gradient is an extremely poor substitute for a Na^+^ gradient in powering transport by VcINDY [8], our data reveal that Li^+^ and Na^+^ stabilise VcINDY equally well, indicating that the same number of bonds are formed, and suggesting identical binding to the *apo* protein. However, our data indicate that, compared with Na^+^, Li^+^ is unable to support efficient succinate binding, with much higher concentrations of succinate required to induce protein stabilisation (Figure 3E). These data suggest that allosteric changes to nearby residues induced by Na^+^ coordination, that cannot be replicated by coordination of Li^+^, are the trigger for succinate binding, and not the mere presence of positively charged ions in the binding site, as previously proposed [13]. This suggestion of allosteric changes to nearby residues is supported by a recent study from our group revealing that VcINDY undergoes Na^+^-specific conformational changes to the hairpins loops that contribute to the binding site [56]. However, a clear understanding of the role of Na^+^ binding on substrate interactions requires an apo structure of VcINDY and further detailed characterisation of this interplay.

### Ligand binding to VcINDY is largely dependent on a molecular ruler-like filter

To understand substrate-binding site flexibility, structurally rationalise DASS:ligand interactions, and unearth interesting interactors that may aid structural studies or inhibitor design, we combined our structural knowledge for VcINDY with a high-throughput thermostability-based interaction screen.

The primary requirement we have identified from our screen of 71 different (mostly anionic) compounds is that successful binding requires the ligand to contain at least two carboxyl groups. The crystal structures of succinate-bound VcINDY reveal that the carboxyl groups of this C_4_-dicarboxylate interact with two regions of the binding pocket; the side-chain amide of N151 and the side-chain hydroxyl of T152 on one side, and the side-chain hydroxyl of S377 and the side-chain amide of N378 on the other (Figure 2C). For successful anion binding, the two carboxyl groups must be positioned similarly to succinate's carboxyl groups in the crystal structures [12, 13], allowing them to contact these critical carboxyl binding regions. The two carboxylate binding regions are essential for successful interaction, demonstrated by the inability of monocarboxylates to stabilise the protein, and largely inflexible, evidenced by the rejection of any dicarboxylate too small (e.g. malonate), too large (e.g. pimelate), or too rigid (e.g. maleate) to successfully bridge them. Therefore, our data suggest VcINDY's anion selectivity is largely governed via a ‘Goldilocks’ molecular ruler principle, in which the dicarboxylates need to be the correct distance in order to bind (they can be neither too long nor too short). The residues that contribute to the carboxyl binding regions are well conserved in the DASS family and the dicarboxylate length dependence has been observed in transport competition assays for other DASS transporters [24, 52], suggesting that this length-dependent selectivity is a general feature of the DASS family. Our understanding of the mechanism of transport by human DASS transporters, and effects of disease causing mutations, has recently been substantially advanced by the publication of the structure of the Na^+^/citrate transporter, NaCT, in the presence of its substrate, citrate, and in the presence of an inhibitor, PF2 [11]. The structure of NaCT revealed that it has the same overall architecture as VcINDY, and the locations of the critical binding site SNT motifs and the location of the Na1 and Na2 sites are conserved between NaCT and VcINDY [11, 57]. Therefore, this demonstrates that VcINDY remains an excellent model for the DASS family from which to derive general mechanistic insight, and it is highly likely that the same length-dependent selectivity between the two carboxylate binding regions that we predict for VcINDY holds true for NaCT as well.

While this interaction screen of VcINDY has provided mechanistic insight and identified novel binders, including TCEP, pyrophosphate, isophthalate, glutarate, icatonate, 5-sulfosalicylate, 1,3,5-benzenetricarboxylate, and tartarate, many questions remain, such as, the structural basis of these protein:ligand interactions. Of particular interest are examples where small changes to a ligand leads to substantial differences in the level of interaction, for example, citrate and tricarballylate. In addition, the assays used here are not able to discern whether the ligands assayed bind to the structurally characterised inward-facing state binding site [12, 13], a binding site formed in a different conformation (e.g. the outward-facing state), or both. Future structural studies with the ligands identified will be essential to ascertain the precise nature of their interactions with VcINDY. In addition, while this thermal shift assay detects potential protein:ligand interactions in a high-throughput, cost-effective manner, it is unsuitable to determine precise binding affinities without substantial modifications that bring their own caveats [58]. Therefore, other methods need to be employed to illuminate this aspect of these interactions. Finally, the thermal shift assay does not address whether an interacting compound is a transportable by VcINDY or not. Determining which of the interacting compounds is not transportable may prove useful for inhibitor development and could reveal mechanistic insight into what is required to trigger transitions between conformations in the transport cycle.

### Lipid interactions appear to alter in response to VcINDY's substrate bound state

The influence of lipids on the mechanism of transport proteins is currently not well understood. However, it is becoming increasingly apparent that specific protein:lipid interactions likely have a profound influence on the structural organisation of transporters and their functional regulation [59–62]. While it has been shown, using molecular dynamic simulations, that the elevator-like mechanism of DASS transporters causes gross deformation of the lipid bilayer during the transport cycle [63], the effects of specific lipids has not been explored. Here, we report the first glimpse into the lipid dependence of DASS transporters, revealing a complex picture.

In this study, our initial attempts to monitor ligand interactions in the presence of lipids using the standard CPM-based thermal shift assay were not possible due to lipid-induced increase in background CPM fluorescence (Supplementary Figure S7). While CPM is considered to be particularly useful for thermal shift assays with membrane proteins because it is weakly fluorescent until conjugated to cysteine residues, our data suggest that CPM's fluorescence also increases in the presence of hydrophobic compounds and not just through conjugation to cysteine, which is supported by the previous observation that cysteine residues are not a pre-requisite for CPM thermal shift assays [64]. We instead turned to a GFP-based stability assay, GFP-TS, to assess the impact of lipids on VcINDY stability. Direct comparison of data derived from CPM and GFP-based assays revealed very similar results, indicating that both methods were monitoring the same protein unfolding events (Figure 7B), and both approaches reported an increase in stability upon inclusion of succinate (Figures 2A and 7C).

Using GFP-TS, we assessed the stabilising effects of added lipids on VcINDY in the absence of the substrate, succinate (Figure 8A). Interestingly, addition of *E. coli* polar lipid extract, which is able to support functional activity of VcINDY [20], had a substantial destabilising effect on the protein compared with the absence of additional lipids (Figure 8A). We found this EPL-induced destabilisation surprising given that the addition of lipids is an oft-used stratagem to *stabilise* membrane proteins for structural studies [43]. Detergent extraction of integral membrane proteins from the lipid bilayer is thought to increase protein dynamics, resulting in destabilisation [43, 65]. Therefore, it is possible that our observed EPL-induced destabilisation is caused by a lipid-based increase in VcINDY dynamics, which we found could be compensated for by the addition of succinate, which may add structural rigidity (Figure 8B) Alternatively, this destabilisation could be caused by the addition of EPL displacing stabilising contacts formed between the micellar detergent and VcINDY. Investigating the stabilising effects of the component parts of EPL revealed some further unexpected results, with PE having a destabilising effect, and both PG and CA significantly stabilising VcINDY (Figure 8A). CA, in particular, plays a clear structural role in several proteins [60, 61], and has been shown to increase conformational rigidity, mechanical stability, and thermostability of other integral membrane proteins [66–68]. As PE is the major lipid species in EPL (∼67%), the substantial PE-induced destabilisation is likely the same phenomenon observed in the EPL-induced destabilisation (Figure 8A).

Addition of succinate to the reactions leads to differential effects in the presence of different lipids. Despite destabilising VcINDY in Na^+^-only conditions, the combination of PE and succinate leads to significantly greater stabilisation than the presence of succinate with no added lipid (Figure 8C), suggestive of a synergistic effect of these interactions, that is, the presence of succinate increases the stabilising effects of PE, or vice versa (Figure 8B,C). Here, we tentatively speculate that the Na^+^/succinate bound state of VcINDY interacts more favourably with PE than the Na^+^ only-bound state. While PG stabilised VcINDY in the Na^+^-only conditions, the addition of succinate induced no further change in stability, implying no interaction with succinate under these conditions (Figure 8B,C). This lack of succinate-induced effect has a few potential explanations, including; interaction with PG discourages succinate interaction by stabilising an occluded conformation of VcINDY in which binding site access is blocked; interaction between VcINDY and anionic PG result in high local negative charge, which shields the electrostatic attraction between anionic succinate and the positively charged binding site; or, hypothetically, binding of PG and succinate impart the same level of stabilisation, and interaction with succinate replaces the interactions with PG, resulting in no net stability change.

The addition on CA to Na^+^-bound VcINDY had, by far, the greatest stabilising effect, even more so than the effect of Na^+^/succinate addition (Figure 8). However, contrary to the effects of succinate addition to all other conditions tested, the addition of succinate to the VcINDY:CA mixture resulted in substantial destabilisation of the protein. Comparing the relative stability in the presence of CA and the absence of additional lipids (Figure 8B), it seems that the presence of succinate abolishes the substantial CA-derived stabilisation suggesting that when bound to Na^+^ and succinate, VcINDY no longer interacts with CA.

Due to the complexity of the lipid environment, the paucity of understanding of how lipid interactions influence membrane protein dynamics and function, and the difficulty inherent in extracting individual effects from an all-encompassing variable like thermostability, it is difficult to draw firm conclusions as to how this apparent interplay would affect VcINDY's transport mechanism. Indeed, despite the support in the literature that GFP-TS can report on protein:lipid interactions [43], caution needs to be taken in interpreting these data due to the unknown effects the different lipids may have on the detergent micelle stability, and the possibility that the lipids could be forming non-specific interactions with the protein. However, these data raise two very interesting possibilities to direct future studies; the presence of different lipids substantially influences VcINDY's substrate interactions perhaps by stabilising a particular conformation; or, subsets of lipids interact preferentially with VcINDY in a particular substrate-bound state, i.e. some lipids bind only to the Na^+^ bound state (PG and CA), while others only interact in the presence of Na^+^ and succinate (PE).

While these data suggest that the binding of substrate has a substantial effect on lipid interactions, which indicates that lipid interactions are not static during transport, the structural basis and the mechanistic implications of this interplay are yet to be determined. However, the potential for a dynamic, regulatory role of lipids in the transport mechanism is supported by the recent elucidation of Na^+^-only bound state of VcINDY, which revealed density for a bound lipid in between the scaffold and transport domains that was not present in the structure containing both Na^+^ and succinate [4]. This lipid binding site would likely be disrupted upon transition between conformations, suggesting a direct link between lipid binding and the substrate bound condition. Lipid and lipid-like density has been observed in similar positions straddling the scaffold and transport domain interface in other elevator transporters (Glt_Ph_, NapA, ASCT2, and EAAT1) [61, 69–71], suggesting that a regulatory role for lipids in transport mechanism may be widespread.

Using thermostability-based assays, this work has furthered our understanding of the interactions between VcINDY, its substrates, and the lipid environment in which it resides. Our data increases our understanding of the Na^+^:substrate interactions for VcINDY, and has identified clear and plausible principles for ligand binding, along with several unexpected ligands which may aid in the design of future DASS family inhibitors. We have investigated the stabilising effects of lipids on VcINDY in different substrate bound conditions, hinting at the potential for dynamic interplay of lipids in the regulation of the DASS mechanism, which is supported by recent structural information from other elevator-like transporters.

## Data Availability

All data are contained within the manuscript.

## Open Access

Open access for this article was enabled by the participation of University of Kent in an all-inclusive *Read & Publish* pilot with Portland Press and the Biochemical Society under a transformative agreement with JISC.

## Abbreviations

CA: cardiolipin
CV: column volumes
DASS: divalent anion sodium symporter
GFP-TS: GFP-based thermostability
PB: purification buffer
PE: phosphatidylethanolamine
PG: phosphatidylglycerol
POPC: 1-palmitoyl-2-oleoyl-glycero-3-phosphocholine
TCEP: tris (2-carboxyethyl)phosphine
