# Supplementary material for: Thermostability-based binding assays reveal complex interplay of cation, substrate and lipid binding in the bacterial DASS transporter, VcINDY
Source: Biochem J. 2021 Nov 9;478(21):3847–67. doi: 10.1042/BCJ20210061 (PMC8652582; doi:10.1042/BCJ20210061)
Supplement: Supplementary Figures S1-S8 [file BCJ-478-3847-s1.pdf]

Supplementary figure legends

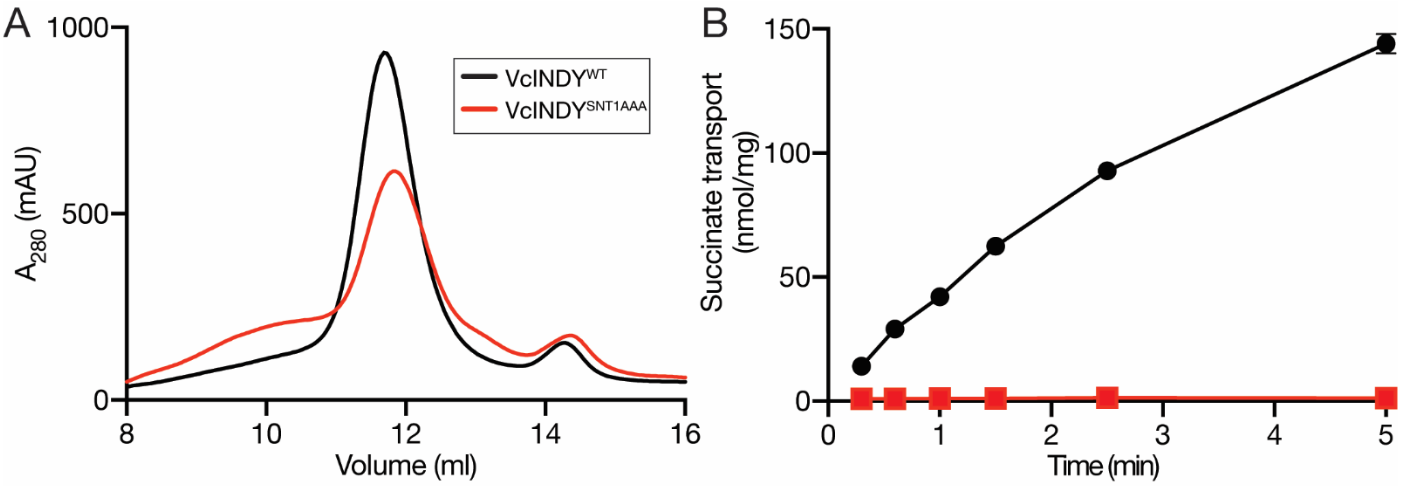

Supplementary figure 1. VcINDY^{SNT1AAA} is stable but incapable of catalysing Na^{+}-driven succinate transport. A) Size exclusion chromatogram of VcINDY^{WT} (black data) and VcINDY^{SNT1AAA} (red data). **B)** Na^{+}-driven [^{3}H]-succinate transport activity of VcINDY^{WT} (black data) and VcINDY^{SNT1AAA} (red data) after purification and reconstitution into proteoliposomes. Data are an average of a triplicate dataset and the error bars represent SEM. Error bars not visible are smaller than the data point symbol.

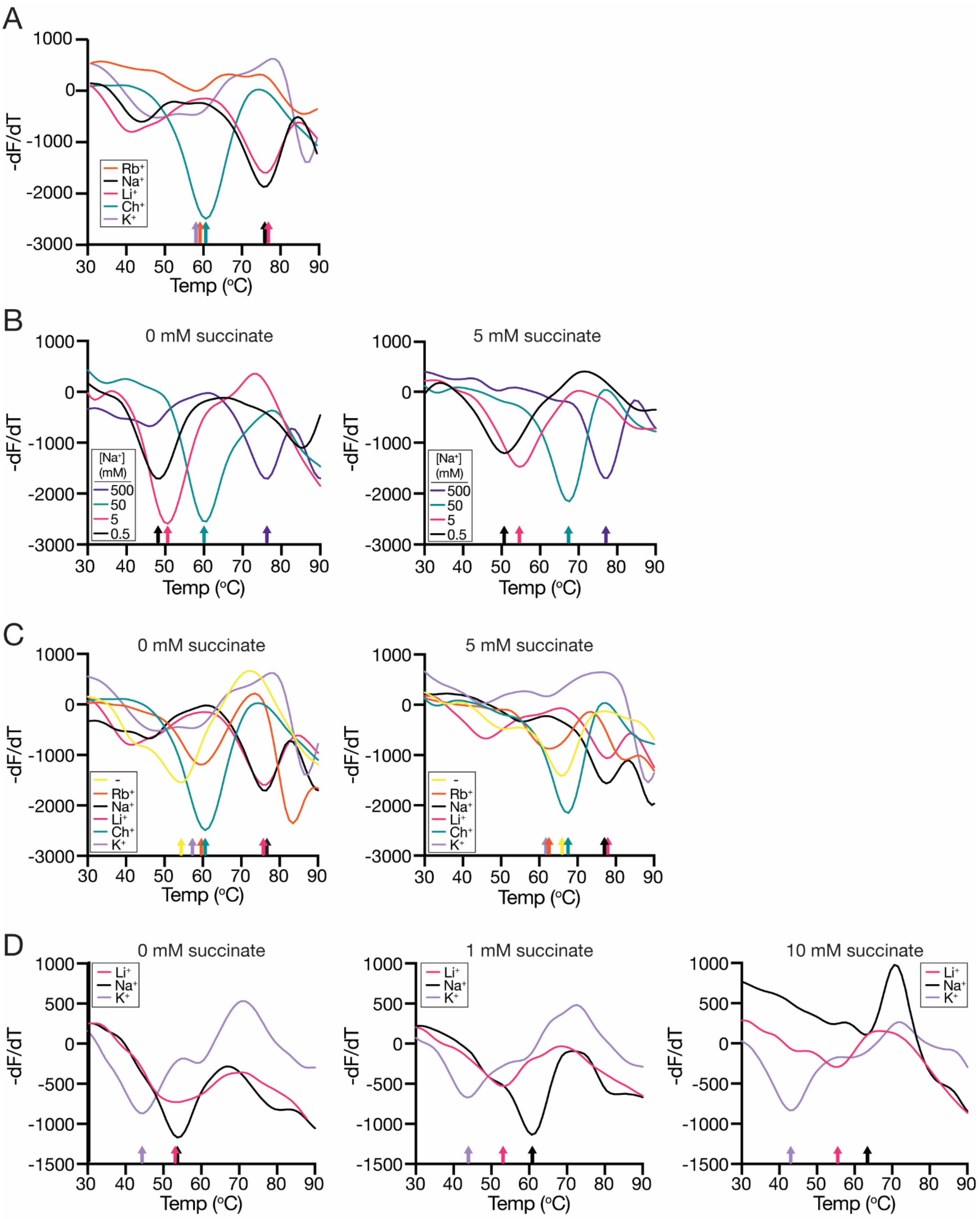

Supplementary figure 2. Representative derivative plots for the cation interactions with VcINDY. Representative derivative plots for VcINDY in the presence of; **(A)** 500 mM Rb^+ (orange), Na^+ (black), Li^+ (pink), choline (green) and K^+ (purple); **(B)** 0.5 mM Na^+ (black), 5 mM Na^+ (pink), 50 mM Na^+ (green) or

500 mM Na^+ (purple) in the absence (left panel) or presence (right panel) of 5 mM succinate; **(C)** 50 mM Na^+ plus 450 mM Rb^+ (orange), Na^+ (black), Li^+ (pink), choline (green), K^+ (purple), or in in the absence of additional cation (yellow), in the absence (left panel) or presence (right panel) of 5 mM succinate; **(D)** 50 mM Na^+ (black), Li^+ (pink), or K^+ (purple) in the absence of succinate (left) or in the presence of 1 mM (middle) or 10 mM (right) succinate. Colour coded arrows on x-axis indicate Tm under that particular condition.

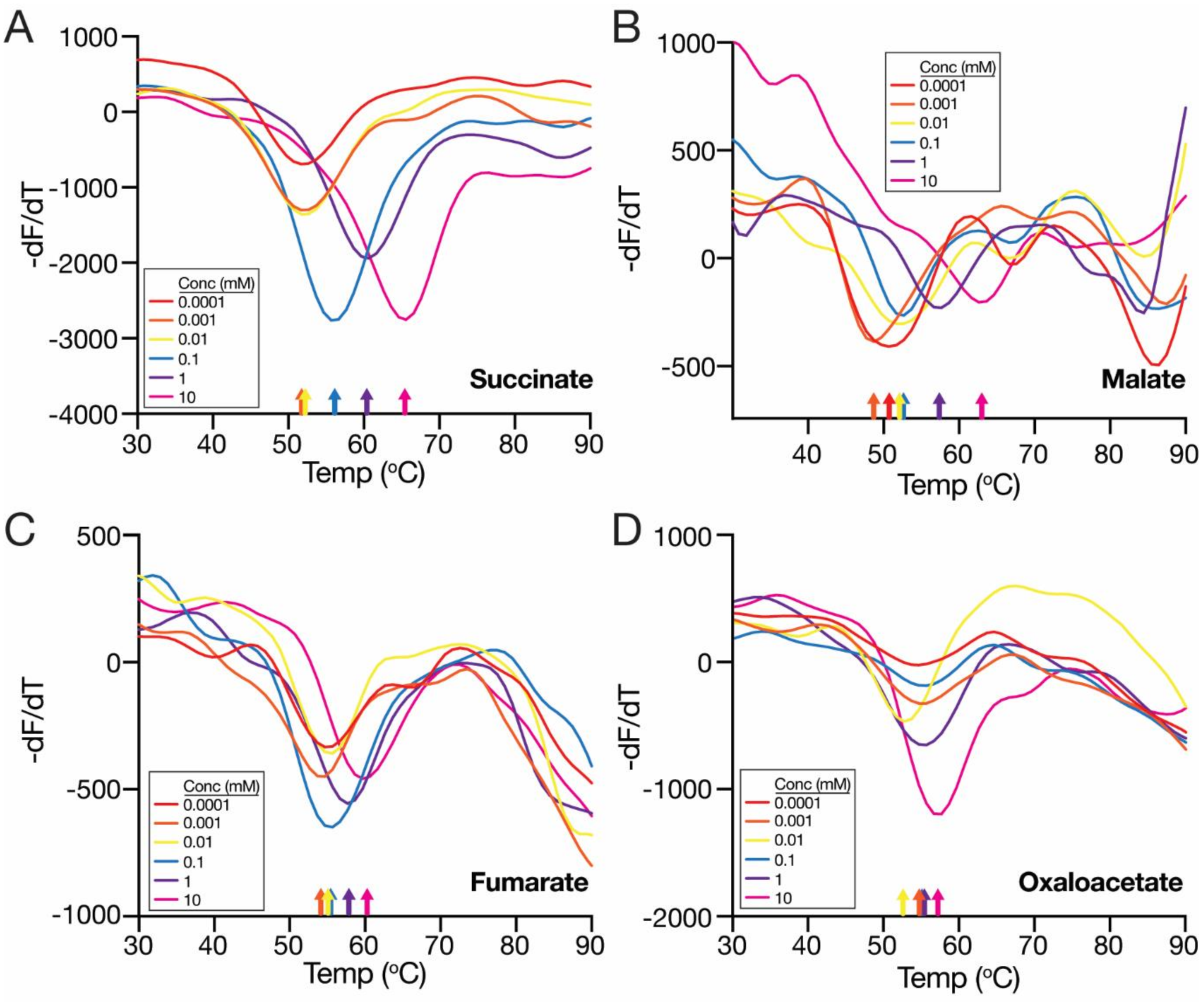

Supplementary figure 3. Representative derivative plots of VcINDY unfolding curves in the presence of increasing concentrations of known substrates. Representative derivative plots of VcINDY unfolding curves in the presence of 50 mM Na^+ plus 0.1 µM (red), 1 µM (orange), 10 µM (yellow), 0.1 mM (blue), 1 mM (purple) or 10 mM (pink) of **(A)** succinate, **(B)** Malate, **(C)** Fumarate, or **(D)** Oxaloacetate. Colour coded arrows on x-axis indicate Tm under that particular condition.

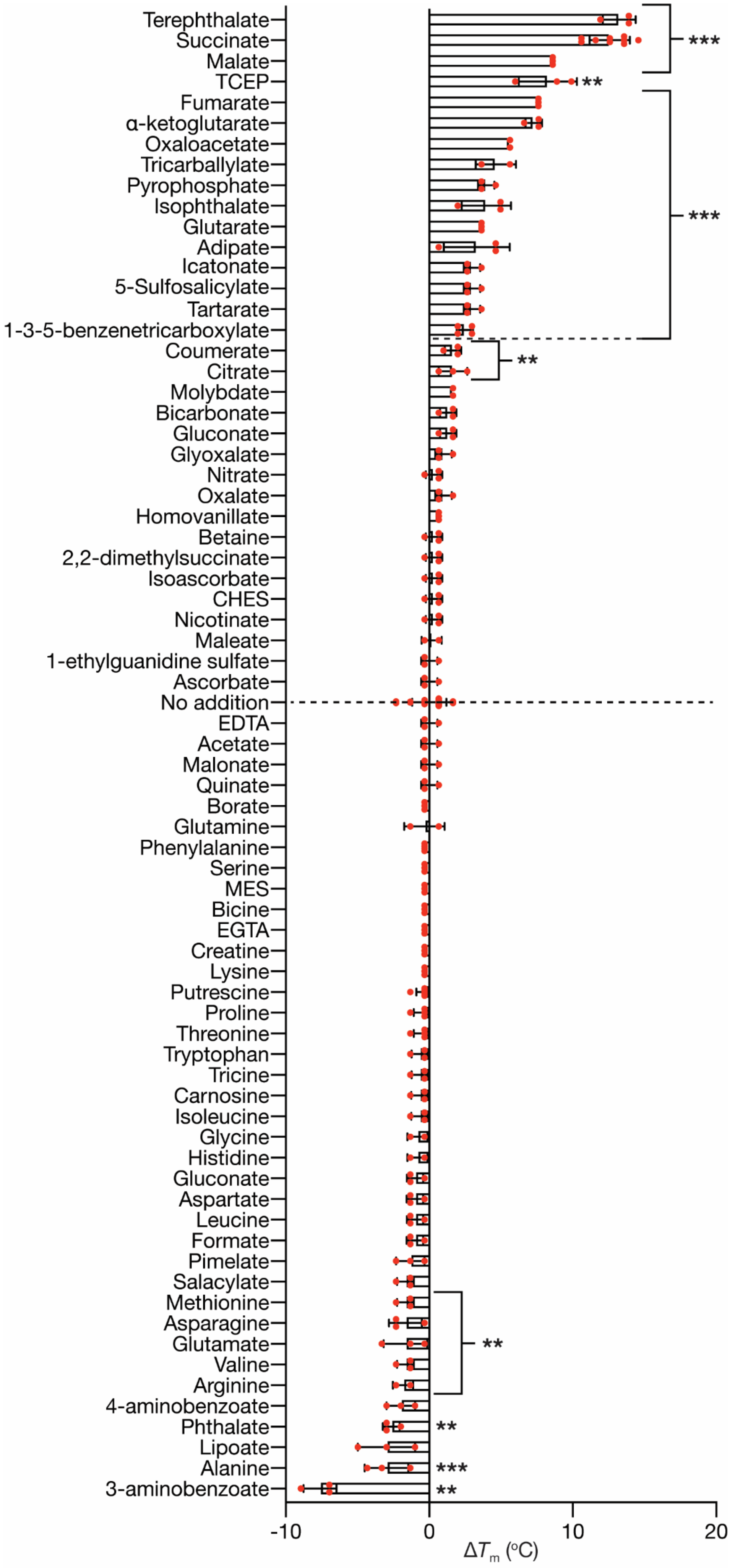

Terephthalate
Succinate
Malate
TCEP
Fumarate
α-ketoglutarate
Oxaloacetate
Tricarballylate
Pyrophosphate
Isophthalate
Glutarate
Adipate
Icatonate
5-Sulfosalicylate
Tartarate
1-3-5-benzenetricarboxylate
Coumerate
Citrate
Molybdate
Bicarbonate
Gluconate
Glyoxalate
Nitrate
Oxalate
Homovanillate
Betaine
2,2-dimethylsuccinate
Isoascorbate
CHES
Nicotinate
Maleate
1-ethylguanidine sulfate
Ascorbate
No addition
EDTA
Acetate
Malonate
Quinate
Borate
Glutamine
Phenylalanine
Serine
MES
Bicine
EGTA
Creatine
Lysine
Putrescine
Proline
Threonine
Tryptophan
Tricine
Carnosine
Isoleucine
Glycine
Histidine
Gluconate
Aspartate
Leucine
Formate
Pimelate
Salacylate
Methionine
Asparagine
Glutamate
Valine
Arginine
4-aminobenzoate
Phthalate
Lipoate
Alanine
3-aminobenzoate
***
**
***
**
**
**
***
**
-10
0
10
20
ΔTm (ºC)

Supplementary figure 4. Large-scale screen of anionic compounds for their ability to stabilise VcINDY. Succinate-induced stabilisation was measured in the presence of 50 mM Na^+. Data are organised by the compounds that increased stability at the top and the compounds that induced destabilisation at the bottom. Dashed line indicates the position of the no substrate ("no addition) control. The average value from at least duplicate datasets are shown, individual datapoints are shown in red, and error bars represent SD. Significance was determined using a 2-sided t-test. $^{**}p \leq 0.05$, $^{***}p \leq 0.001$.

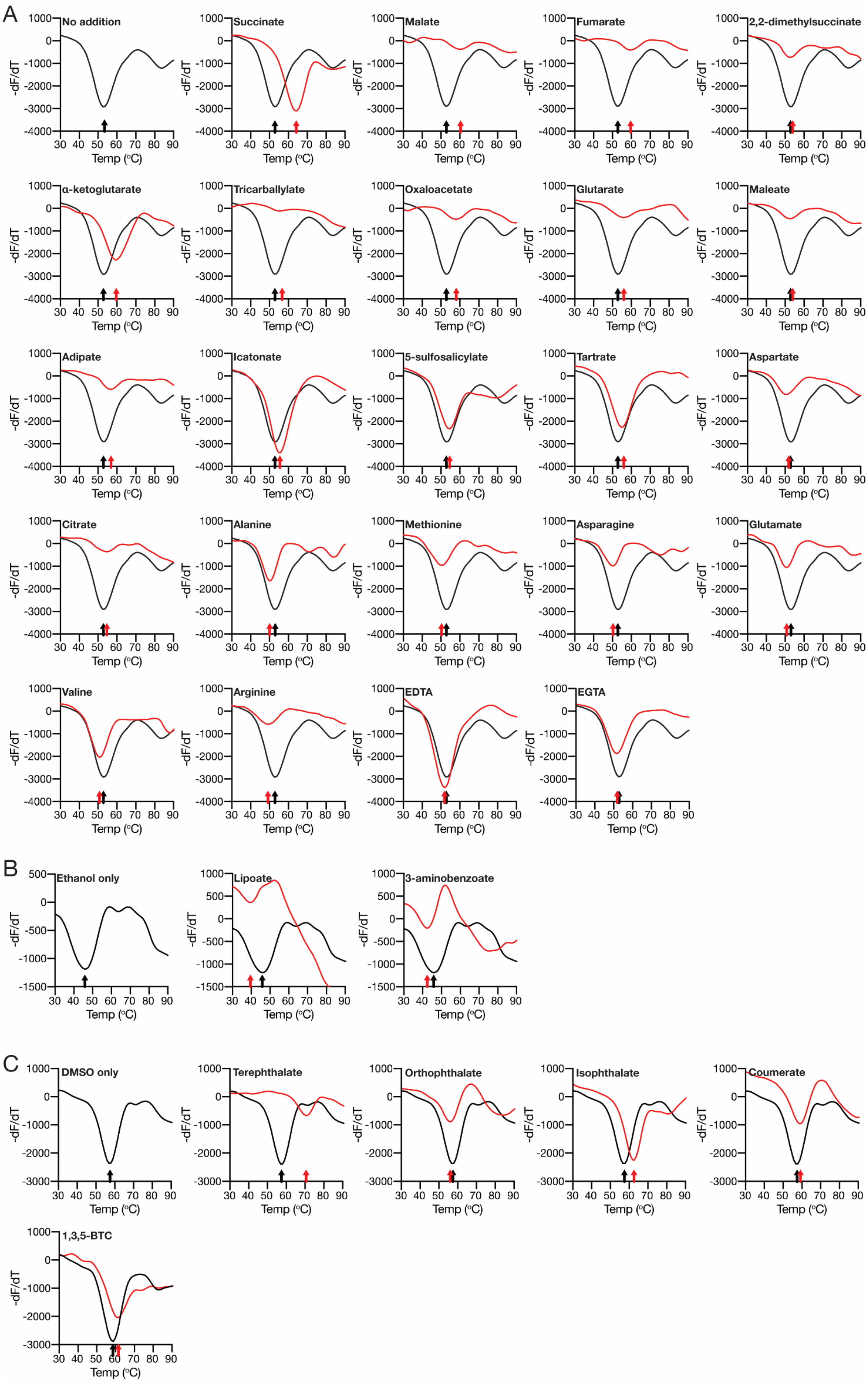
A
No addition
Succinate
Malate
Fumarate
2,2-dimethylsuccinate
α-ketoglutarate
Tricarballylate
Oxaloacetate
Glutarate
Maleate
Adipate
Icatonate
5-sulfosalicylate
Tartrate
Aspartate
Citrate
Alanine
Methionine
Asparagine
Glutamate
Valine
Arginine
EDTA
EGTA
B
Ethanol only
Lipoate
3-aminobenzoate
C
DMSO only
Terephthalate
Orthophthalate
Isophthalate
Coumerate
1,3,5-BTC
-dF/dT
Temp (°C)

Supplementary figure 5. Representative derivative plots of the unfolding curve of VcINDY in the presence of various compounds. Representative derivative plots of VcINDY unfolding curves in the presence of all compounds that had a significant effect on the Tm of VcINDY^{WT}. Substrates are grouped depending on whether they were dissolved in **(A)** water, **(B)** ethanol, or **(C)** DMSO. Black traces are the derivative plots in the absence of substrate (but in the presence of vehicle), and red traces are in the presence of substrate. Colour coded arrows on the x-axis indicate Tm under that condition.

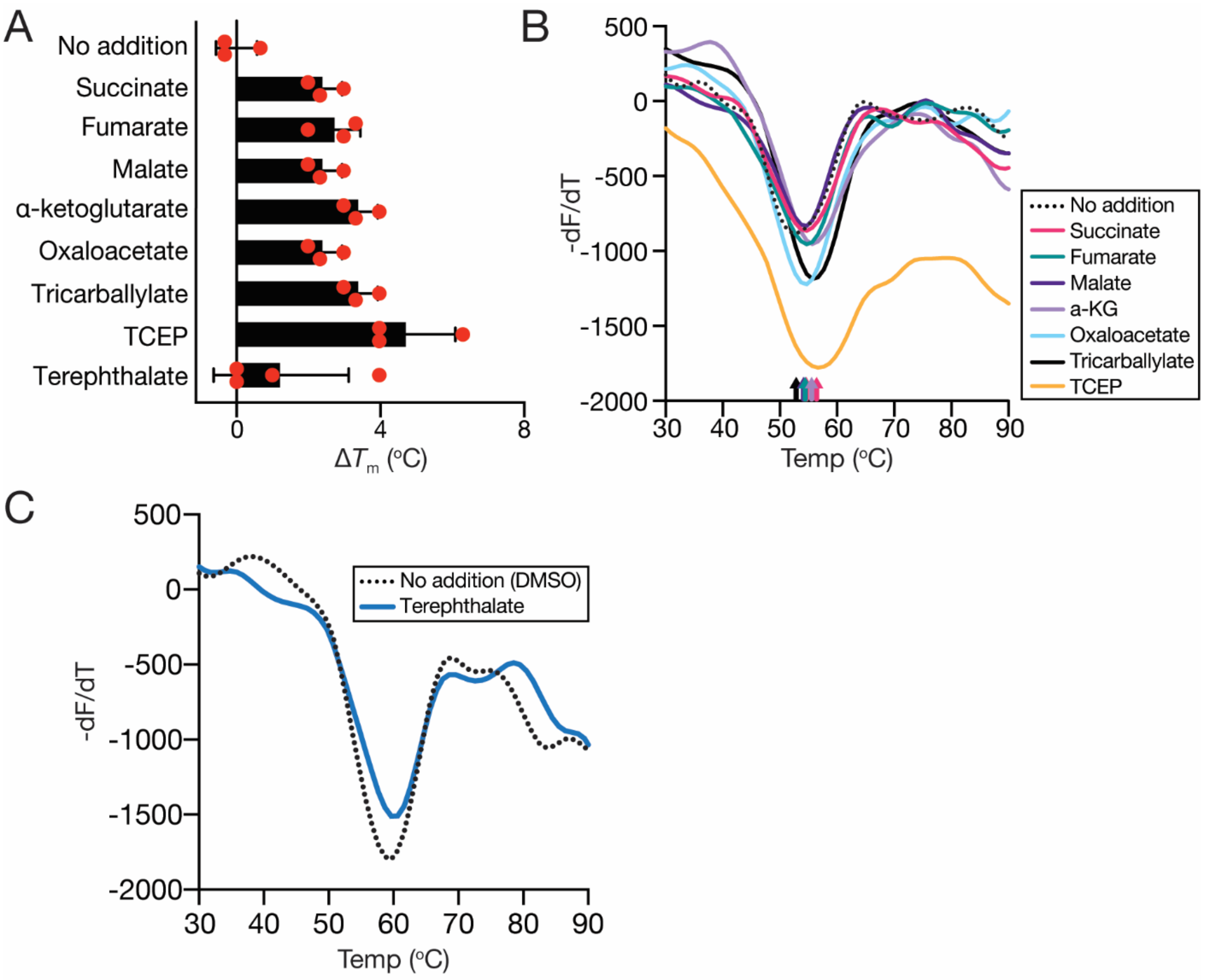

Supplementary figure 6. Thermostabilisation effects of select ligands on VcINDY^{SNT1AAA} binding site mutant. A) The melting temperature shift (ΔTm) of VcINDY^{SNT1AAA} in the presence of 50 mM NaCl and 10 mM of the indicated compound compared to the absence of added ligand. Data are the average of at least 3 data points, error represents SD, and red circles are individual datapoints. **B)** Representative derivative plots for VcINDY in the absence of added ligand or in the presence of indicated water-soluble compound. **C)** Representative derivative plots for VcINDY in the absence of added ligand (but in the presence of DMSO) or in the presence of terephthalate dissolved in DMSO.

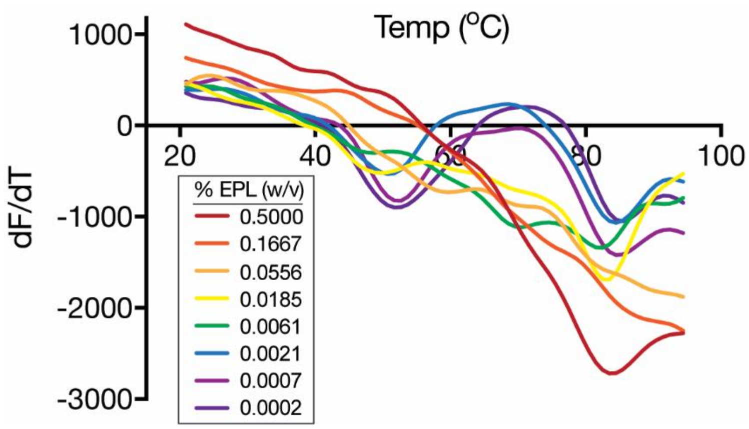

Supplementary figure 7. Increasing lipid content obscures with CPM-based melt curve for VcINDY. Derivative melt curve for VcINDY^{WT} in the presence of increasing concentrations of *E. coli* polar lipids (EPL). The Tm for VcINDY in the absence of lipids is ~54°C.

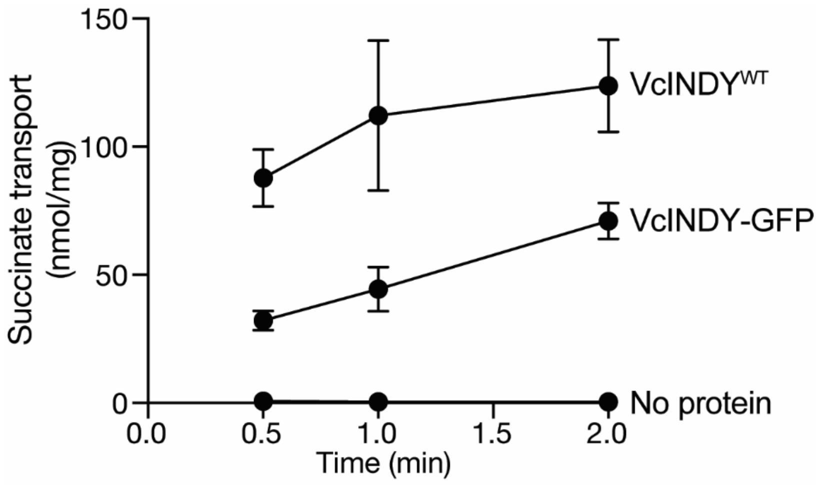

Supplementary figure 8. VcINDY-GFP has robust transport activity. [^{3}H]-succinate transport activity of proteoliposomes containing no protein, VcINDY^{WT}, or VcINDY-GFP fusion. Data shown is the average of triplicate data sets and error bars represent SD. Error bars not visible are smaller than the data point symbol.
